# Targeting BCR-ABL^+^ stem/progenitor cells and BCR-ABL-T315I mutant cells by effective inhibition of the BCR-ABL-Tyr177-GRB2 complex

**DOI:** 10.18632/oncotarget.18216

**Published:** 2017-05-25

**Authors:** Min Chen, Ali G. Turhan, Hongxia Ding, Qingcong Lin, Kun Meng, Xiaoyan Jiang

**Affiliations:** ^1^ Terry Fox Laboratory, British Columbia Cancer Agency and Department of Medical Genetics, University of British Columbia, Vancouver, BC, Canada; ^2^ Department of Hematology, Paris Sud University Hospitals, University Paris Sud 11 and INSERM U935, Villejuif, France; ^3^ Shenogen Pharma Group Ltd, Beijing, China

**Keywords:** BCR-ABL+ human leukemia, leukemic stem cells, tyrosine kinase inhibitors, Icaritin (SNG162 and SNG1153), ERα36

## Abstract

Treatment of BCR-ABL^+^ human leukemia has been significantly improved by ABL tyrosine kinase inhibitors (TKIs), but they are not curative for most patients and relapses are frequently associated with BCR-ABL mutations, warranting new targets for improved treatments. We have now demonstrated that protein expression of human estrogen receptor alpha 36 (ERα36), an alternative splicing variant of human estrogen receptor alpha 66 (ERα66), is highly increased in TKI-insensitive CD34^+^ chronic myeloid leukemia (CML) cells and BCR-ABL-T315I mutant cells, and is abnormally localized in plasma membrane and cytoplasm. Interestingly, new pre-clinically-validated analogs of Icaritin (SNG162 and SNG1153), which target abnormal ERα36 activity, inhibit cell growth and induce apoptosis of BCR-ABL^+^ leukemic cells, particularly BCR-ABL-T315I mutant cells. A combination of SNG inhibitors and TKI selectively eliminates treatment-naïve TKI-insensitive stem/progenitor cells while sparing healthy counterparts. Oral TKI dasatinib combined with potent SNG1153 inhibitor effectively eliminates infiltrated BCR-ABL^+^ blast cells and enhances survival of mice. Importantly, a unique mechanism of SNG inhibition was uncovered by demonstrating a marked interruption of the BCR-ABLTyr177-GRB2 interaction, leading to inhibition of the downstream RAS/MAPK pathway. This new combination therapy may lead to more effective disease eradication, especially in patients at high risk of TKI resistance and disease progression.

## INTRODUCTION

Chronic myeloid leukemia (CML) is a myeloproliferative disease that originates from hematopoietic stem cells and evolves through three stages: chronic phase (CP), accelerated phases (AP) and blast crisis (BC). BCR-ABL^+^ acute lymphoblastic leukemia (ALL) closely resembles the aggressive lymphoid BC of CML and is prone to relapse with current therapies [1–5]. Both are uniquely defined by clone-specific BCR-ABL fusion genes that encode oncoprotein isoforms (p210^BCR-ABL^ and p190^BCR-ABL^) with constitutively elevated tyrosine kinase (TK) activity, driving pathogenesis [6, 7], which perturb many signaling pathways, including RAS/MAPK, PI3K/AKT and JAK2/STAT5 pathways [6, 7]. Particularly, the regulatory domains of BCR, such as tyrosine residue 177 (Tyr177), can be phosphorylated by ABL. This allows for binding with GRB2, growth factor receptor-bound protein 2, through its SRC Homology 2 (SH2) domain, an essential interaction for BCR-ABL-mediated leukemogenesis and RAS/MAPK activation [8–12].

The small molecule inhibitor Imatinib (IM) has been the first line treatment for CP-CML patients for a decade, with remarkable efficacy [13, 14]. However, it is much less effective for treatment of advanced CML and BCR-ABL^+^ ALL patients, and primary and acquired IM resistance remain problematic [5, 15–17]. In particular, relapses are frequently associated with point mutations in the BCR-ABL TK domain, with more than 100 mutations documented [18–21]. Second generation TKIs, such as dasatinib (DA) and nilotinib (NL), have increased potency and show a broader spectrum of activity against mutant forms of BCR-ABL [22–24]. However, they cannot target a critical T315I gatekeeper mutation of BCR-ABL in TKI-resistant patients [17, 23, 25]. The third generation TKI ponatinib has been reported to inhibit the T315I mutation, but it displays toxicity and a phase 2 clinical trial was discontinued [26, 27]. In addition, most patients harbor residual leukemic stem cells (LSCs), which are known to be genetically unstable and less responsive to TKI treatments [19–21, 28–30]. These observations emphasize the need to develop new therapeutic agents and combination strategies to specifically target LSCs and BCR-ABL-T315I mutant cells.

Estrogen receptor variant ERα36 is highly deregulated in breast and other cancers [31–35]. It has considerable sequence homology with full length ERα66, but lacks the transcriptional activation domains (AF1 and AF2) and contains a unique C-terminal 27 amino acid sequence [36]. Interestingly, its abnormal expression is associated with poor prognosis in breast cancer [33]. In silico modeling shows that ER-α36 protein is structurally unhindered by the lack of a stretch of helical chain present in ERα66, resulting in a more open ligand-binding pocket in ERα36. By screening a library of traditional Chinese medicine compounds, a small molecular inhibitor (Icaritin, SNG162), a key component of Epimedium flavonoid isolated from Epimedium Genus, was identified, which inhibits growth of breast and liver cancer cells [37]. It was initially suggested that Icaritin and its analogs may function as estrogen modulators in regulating breast cell growth, but recent evidence demonstrates that it has broader anti-cancer activity in many cancer types [38–41]. Ongoing SNG162 phase I/II clinical trials have been encouraging for patients with advanced hepatocellular carcinoma and advanced solid tumors (NCT01278810, NCT01972672 and NCT02496949). Interestingly, SNG162 also inhibits the growth of leukemic cell lines, including BCR-ABL^+^ cell lines, possibly by interfering with MAPK/ERK and JAK/STAT3 signaling pathways [42]. However, it is not known whether it has any biological effects on TKI-insensitive stem/progenitor cells or aggressive BCR-ABL^+^ blast cells and the underlying molecular mechanisms are also not understood. Here, we examined the biological effects of highly selective, orally bioavailable SNG inhibitors alone, or in combination with TKIs, on CD34^+^ treatment-naïve IM-nonresponder cells and TKI-resistant cells, including BCR-ABL-T315I mutant cells. We demonstrated that SNG inhibitor alone strongly inhibited cell growth and induced apoptosis; these effects were enhanced synergistically by TKIs *in vitro*. The combination also significantly enhanced survival in mice. Importantly, a unique molecular mechanism for the inhibitory effects on these TKI-resistant cells was discovered: marked disruption of the BCR-ABL-Tyr 177-GRB2 interaction and further inactivation of the downstream RAS-MAPK pathway.

## RESULTS

### Protein expression of ERα36 is highly increased in CD34^+^ CML cells and BCR-ABL-T315I mutant cells and it localizes to plasma membrane and cytoplasm

**Figure 1:**
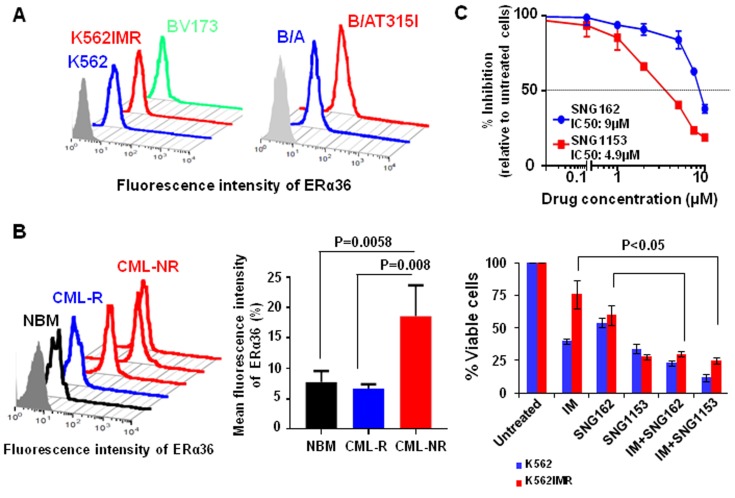
Increased surface expression of ERα36 in TKI-resistant cells and CD34 + IM-nonresponder cells. **A**. Detection of surface expression of ERα36 in parental K562 and K562 IM-resistant cells (K562IMR), BV173 cells and human UT7 cells expressing either wild-type BCR-ABL (B/A) or BCR-ABL-T315 mutant (B/AT315I) cells using a specific anti-ERα36 antibody. **B**. Expression of ERα36 in CD34^+^ cells isolated from IM-nonresponders (*n* = 5), IM-responders (*n* = 3) and normal donors (*n* = 4). The differences detected were shown in mean fluorescence intensity of ERα36 in these samples. Values shown are the mean ± SEM of measurement from normal and CML patients. **C**. IC_50_ curves for K562 cells after 48 hours treatment with SNG162 and SNG1153 (from 0.1μM to 10 μM range). K562 and K562IMR cells were treated with IM (0.5 μM for K562 and 2.5 μM for K562IMR), SNG162 (5 μM) or SNG1153 (2.5 μM) alone or in combination for 48 hours. Viable cells were analyzed by counting trypan blue excluding cells. The percentage of viable cells relative to untreated cells was expressed. Data shown are mean ± SEM of measurements from three independent experiments.

To investigate if expression of ERα36 is altered in CML cells, we compared protein expression of ERα36 in IM-sensitive vs. IM-resistant K562 cells (K562IMR, a spontaneously derived cell line without BCR-ABL mutation) [43], BCR-ABL-transduced human UT7 cells *vs*. BCR-ABL-T315I mutant-transduced cells and BCR-ABL^+^ BV173 blast cells, using FACS analysis. Surface expression of ERα36 is greatly increased in K562IMR cells compared to parental K562 cells, and in BCR-ABL-T315I mutant cells compared to wild-type BCR-ABL-transduced cells (Figure 1A). Notably, the highest surface expression of ERα36 was found in aggressive BCR-ABL^+^ blast cells (BV173) from late stage disease. Interestingly, CD34^+^ CML cells from subsequent IM-nonresponders (*n* = 5) displayed significantly high levels of ERα36 expression compared to CD34^+^ cells from IM-responders (*n* = 3) and NBM cells (*n* = 4, 2-3 fold, *P* < 0.01, Figure 1B). Immunostaining in conjunction with FACS analysis demonstrated that ERα36 is mainly localized to the plasma membrane and cytoplasm, while ERα66 mainly localizes to the nucleus (Figure 1A-1B and Supplementary Figure 1A). Thus, abnormal localization and increased expression of ERα36 occur in IM-nonresponder CML stem/progenitor cells and IM-resistant cell lines, including BCR-ABL-T315I mutant cells.

### SNG162 or SNG1153 inhibitor alone inhibit cell proliferation in CML cells and these effects are enhanced by IM

**Figure 2:**
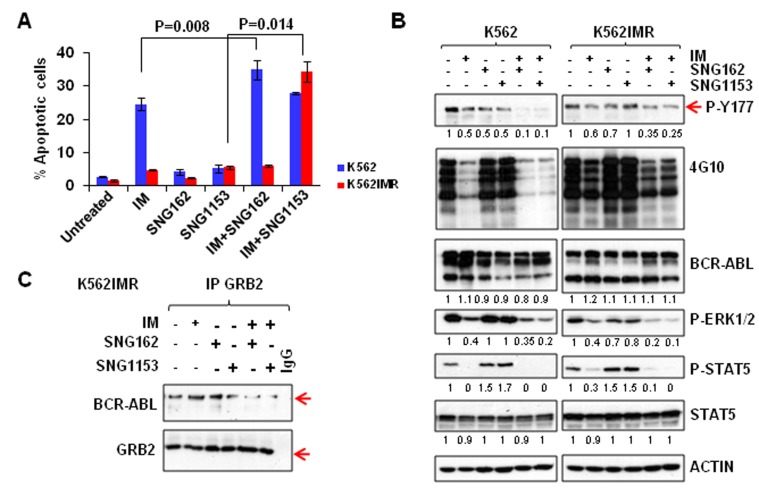
A combination of SNG inhibitors and TKI is more effective in inducing apoptosis and suppressing the phosphorylation of tyrosine 177 of BCR-ABL in K562 and K562IMR cells **A**. K562 and K562IMR cells were treated with IM (0.5 μM for K562 and 2.5 μM for K562IMR), SNG162 (5 μM) or SNG1153 (2.5 μM) alone or in combination for 48 hours. Apoptotic cells were determined by Annexin V^+^ staining. Values are presented as mean ± SEM of three different experiments. **B**. Western blot analysis of protein expression of K562 or K562IMR cells treated with IM or SNG inhibitors, alone or in combination, for 48 hours. Specific antibodies used are indicated. The densitometry values of protein expression changes are indicated as compared to untreated control. **C**. GRB2 was immunoprecipitated from K562IMR cell lysates with the same treatment as indicated in B. The immunoprecipitates were then probed with either BCR-ABL or GRB2 antibodies.

To investigate if suppression of abnormal ERα36 activity can affect proliferation and viability of CML cells, SNG162 inhibitor, and the more potent second generation SNG1153, were used. These molecules were generated based on the drug structure of Icaritin, which was identified by drug screening and can mediate the activity of ERα36 [38, 44]. The IC_50_ values of SNG162 and SNG1153 are 9μM and 4.9μM in K562 cells (Figure 1C). Notably, SNG1153 alone inhibited viability of K562 and K562IMR up to 70% compared to SNG162 (~40%) or IM (55% in K562 cells and 25% in IMR, Figure 1C). As expected, K562IMR cells were resistant to IM-induced apoptosis, with only 5% Annexin V^+^ cells after 48 hours of exposure to IM, while the addition of SNG1153 strongly increased the frequency of Annexin V^+^ cells (*P* = 0.014, Figure 2A). This effect was not observed in K562IMR cells with SNG162 plus IM, suggesting that SNG1153 is a more potent inhibitor, which inhibits cell growth and induces apoptosis of IM-resistant cells.

To determine whether the combination of SNG inhibitors and a TKI had synergistic or addictive effects, viability assays were performed on K562IMR cells, with graded doses of SNG1153 and IM, alone or in combination, for 48 hours. The average CI for ED50, ED75, and ED90 was calculated to be 0.22, indicating that the combination is highly synergistic (Supplementary Figure 1B).

To further determine if knockdown of ERα36 could enhance IM-mediated inhibition of cell proliferation and apoptosis, as demonstrated by SNG inhibitors, K562 cells were transfected with either a non-targeting siRNA control or two different siRNA targeting sequences against human ER (siER1 and siER2). Western blot analysis showed that transfection with siER1 reduced protein expression of ERα36 (~50%), while siER2 suppressed ERα66 (~70%, Supplementary Figure 2A). Interestingly, suppression of ERα36 in K562 cells increased sensitivity towards IM treatment compared to K562 cells transfected with scrambled siRNA or siER2, which suppressed full length ERα66, as assessed by cell viability and apoptosis assays (Supplementary Figure 2A). Thus, genetic and pharmacological suppression of ERα36 reduced proliferation and increased apoptosis of drug-insensitive CML cells, and sensitized them to IM treatment.

### Combination treatment of IM with SNG inhibitors reduces phosphorylation of BCR-ABL-Tyr177 and disrupts its interaction with GRB2 in IM-resistant and BCR-ABL-T315I mutant cells

To investigate the molecular mechanisms of SNG inhibitor regulation of IM-response/resistance of CML cells, we compared protein phosphorylation of key proteins in BCR-ABL-mediated signaling. As expected, BCR-ABL tyrosine kinase activity was reduced by IM in K562 and K562IMR cells and a combination of IM with SNG inhibitors further reduced its activity to some extent (Figure 2B). Most interestingly, phosphorylation of BCR-ABL on tyrosine residue 177 (Tyr177) was substantially reduced in K562 cells, and to a lesser extent in K562IMR cells, by a combination of SNG162 or SNG1153 plus IM (Figure 2B). Co-immunoprecipitation experiments clearly demonstrated that the BCR-ABLTyr177-GRB2 protein interaction was markedly interrupted in K562IMR cells treated with SNG162 or SNG1153 plus IM, which was not observed with IM or SNG inhibitors alone (Figure 2C). This observation was supported by detection of a great reduction in phosphorylation of ERK1/2 kinase, a key component of the RAS-MAPK pathway, by IM plus SNG inhibitors (Figure 2B). In contrast, p-STAT5 was reduced with IM alone.

**Figure 3:**
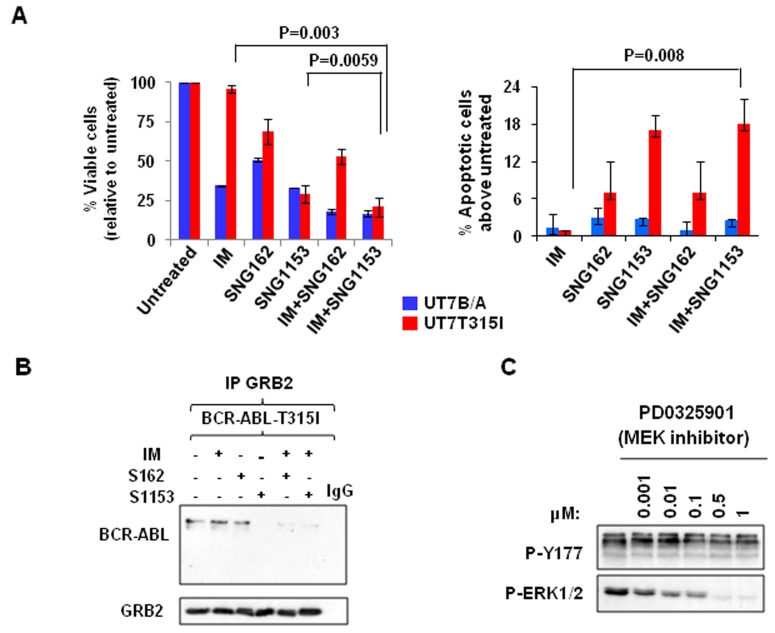
Combination treatment with SNG inhibitors and IM disrupts the BCR-ABL-Tyr177-GRB2 interaction in BCR-ABL-T315I mutant cells **A**. BCR-ABL (UT7B/A) and BCR-ABL-T315I (UT7T315I) cells were treated with IM (1.0μM for BCR-ABL cells and 5μM for BCR-ABL-T315I cells), SNG162 (10μM) and SNG1153 (5μM) alone or in combination for 48 hours and then analyzed with viability and apoptosis assays. **B**. GRB2 was immunoprecipitated and probed with a BCR-ABL or GRB2 antibody in BCR-ABL-T315I cells in the presence or absence of inhibitors indicated. **C**. BCR-ABL-T315I cells were treated with a MEK inhibitor (PD0325901), at concentrations indicated, for two hours, and cells were then harvested for Western blot analysis with indicated antibodies.

To investigate the effects of SNG inhibitors on BCR-ABL-T315I mutant cells, BCR-ABL (wild-type) and BCR-ABL-T315I transduced human UT7 cells were cultured with IM, SNG162, and SNG1153, alone or in combination, and viability assays were conducted. As expected, BCR-ABL-T315I cells were not responsive to IM treatment (96% viable cells), but were highly sensitive to SNG inhibitor treatment; SNG1153 alone effectively inhibited the growth of BCR-ABL-T315I cells up to 70% and strongly induced apoptosis compared to controls (Figure 3A). The strong inhibitory effect of SNG1153 on BCR-ABL-T315I mutant cells was further demonstrated by a pronounced reduction in total colony numbers and colony size in colony-forming cell (CFC) assays (*P* < 0.05, Supplementary Figure 2B). Co-immunoprecipitation demonstrated that SNG1153 alone significantly disrupted the BCR-ABL-GRB2 interaction (Figure 3B). These changes were not observed in BCR-ABL-T315I mutant cells with MEK inhibitor PD0325901 (Figure 3C), suggesting that SNG inhibitors directly affect phosphorylation status of BCR-ABL-Tyr177 and that reduction in phosphor-ERK1/2 kinase is a downstream event.

### SNG inhibitors in combination with TKIs are more effective in eliminating TKI-insensitive CML stem and progenitor cells

**Figure 4:**
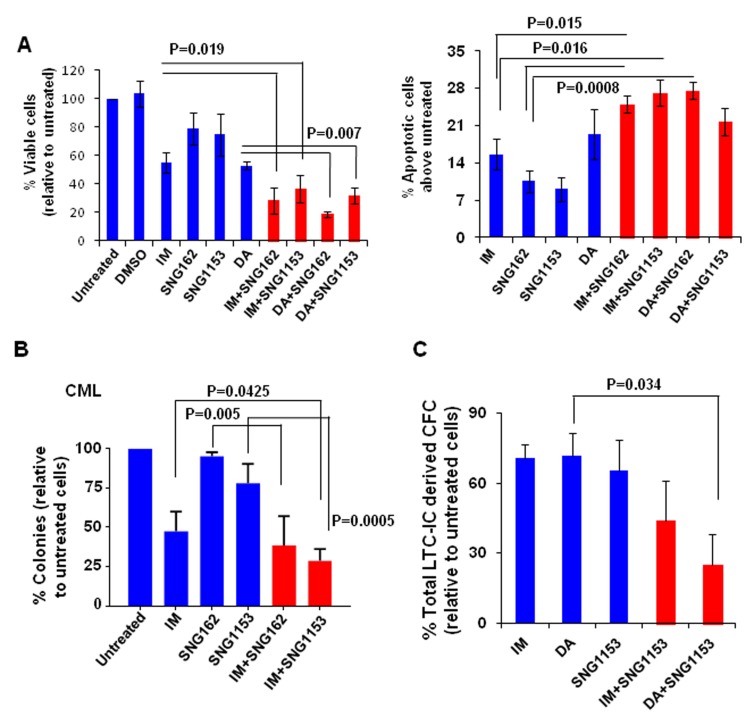
A combination of SNG inhibitors with TKIs is more effective at inhibiting IM-nonresponder stem/progenitor cells **A**. Viability of CD34^+^ CML cells obtained from IM-nonresponders (*n* = 4) was measured 72 hours after these cells were exposed to IM (5μM), DA (100nM), SNG162 (10μM) or SNG1153 (2.5μM), alone or in combination. Apoptosis assays were conducted in the same cells by detection of Annexin V^+^ cells compared to untreated cells. **B**. CFC assays were performed on CD34^+^ cells from IM-nonresponders (*n* = 4) with IM (5μM), SNG162 (10μM) or SNG1153 (5μM), alone or in combination. Colonies were counted after 14 days and were presented as the percentage of total numbers obtained from cells without any inhibitors. **C**. Long-term culture initiating cell assays (LTC-IC) were performed on CD34^+^ CML cells obtained from IM-nonresponders (*n* = 3). The total LTC-IC derived colonies from treatment groups were expressed as a percentage of the LTC-IC derived total CFC numbers obtained from cells without any treatment.

To investigate whether dual treatment may be effective for CML patients who do not respond adequately to single TKI treatment, we performed viability and apoptosis assays on CD34^+^ stem/progenitor cells obtained at diagnosis from CML patients classified retrospectively as IM-nonresponders (*n* = 4, Supplementary Table 1)[45, 46]. As we reported previously [47], only 50% of pre-treated CD34^+^ CML stem/progenitor cells from IM-nonresponders responded to IM or DA treatment, and combination treatments of IM or DA with SNG inhibitors were significantly more effective (*P* < 0.02, Figure 4A). Similarly, the combination significantly increased Annexin V^+^ cells (*P* < 0.02, Figure 4A).

To further determine if SNG inhibitors plus TKIs eliminate pre-treatment LSCs and their progenitor cells from IM-nonresponders, *in vitro* long-term progenitor (CFC) and stem cell assays (LTC-IC) were performed. Interestingly, a combination of SNG162 or SNG1135 with IM significantly inhibited colony growth of CD34^+^ cells compared to single agents (72-80% *vs*. 10-45% reduction, *P* < 0.05, Figure 4B). Furthermore, LTC-IC assays showed that more primitive CML cells were more significantly eliminated by combination treatment, particularly by DA with SNG1153 (*P* = 0.034, Figure 4C), indicating the potential benefit of combination therapy for targeting LSCs. Importantly, both SNG162 (10μM) and SNG1135 (5μM) have no toxicity on CD34^+^ healthy BM cells (*n* = 7) in CFC assays, and the combination of SNG inhibitors and TKIs shows a similar effect to TKI alone (Supplementary Figure 3A).

### SNG inhibitors in combination with TKIs effectively eliminate aggressive BCR-ABL^+^ blast cells *in vitro* and significantly enhance the survival of leukemic mice

**Figure 5:**
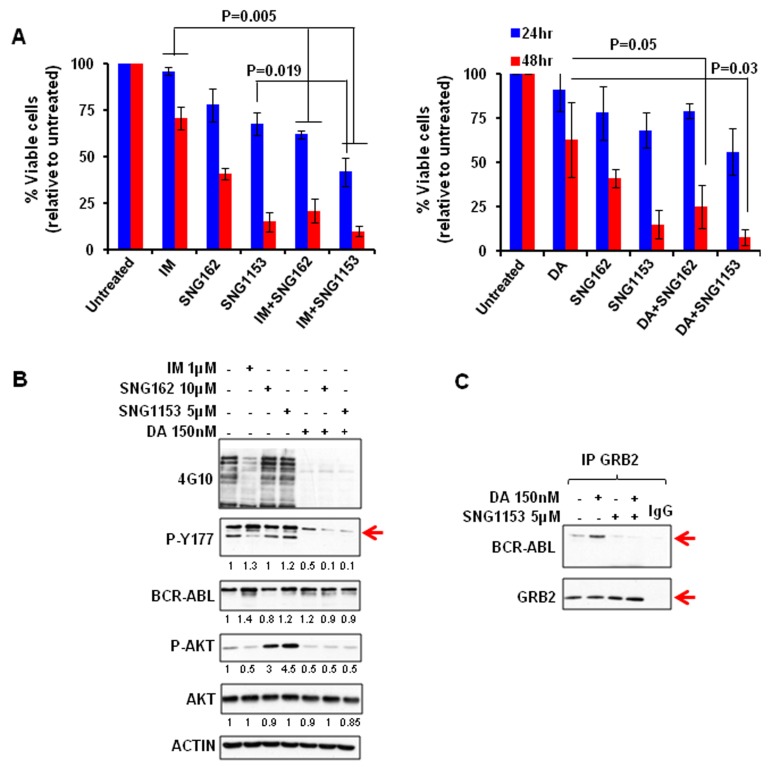
SNG inhibitors in combination with TKIs effectively eliminate aggressive BCR-ABL + blast cells in vitro and disrupt the interaction between BCR-ABL-Tyr177 and GRB2. **A**. BV173 cells were treated with IM (1μM), DA (150nM), SNG162 (10μM), SNG1153 (5μM), alone or in combination, for 24 or 48 hours. The percentage of viable cells under each treatment condition is presented as mean ± SEM. **B**. Whole proteins were extracted from BV173 cells treated with inhibitors for 24 hours, and analyzed by Western blotting or by co-immunoprecipitation analysis. The densitometry values of protein expression changes are indicated as compared to untreated control.

The combination effects of SNG inhibitors with TKIs were further investigated in aggressive BCR-ABL^+^ blast cells (BV173) that expressed the highest levels of ERα36 (Figure 1A), since TKI monotherapy is much less effective in this late stage disease [3, 4]. BV173 cell viability was significantly reduced with SNG162 (60%) and SNG1153 (80%) alone, while IM or DA only resulted in 30% reduction after 48 hours; this was further enhanced by the combination of SNG inhibitors with TKI (*P* < 0.05, Figure 5A). Western blot analysis demonstrated that SNG162 or SNG1135 plus IM or DA reduced phosphorylation of Y177 of BCR-ABL and disrupted the interaction with GRB2 (Figure 5B, IM plus SNG inhibitors not shown). The result was further confirmed in the same cells treated with SNG1135 and DA, alone or in combination, by co-immunoprecipitation experiments (Figure 5C).

**Figure 6:**
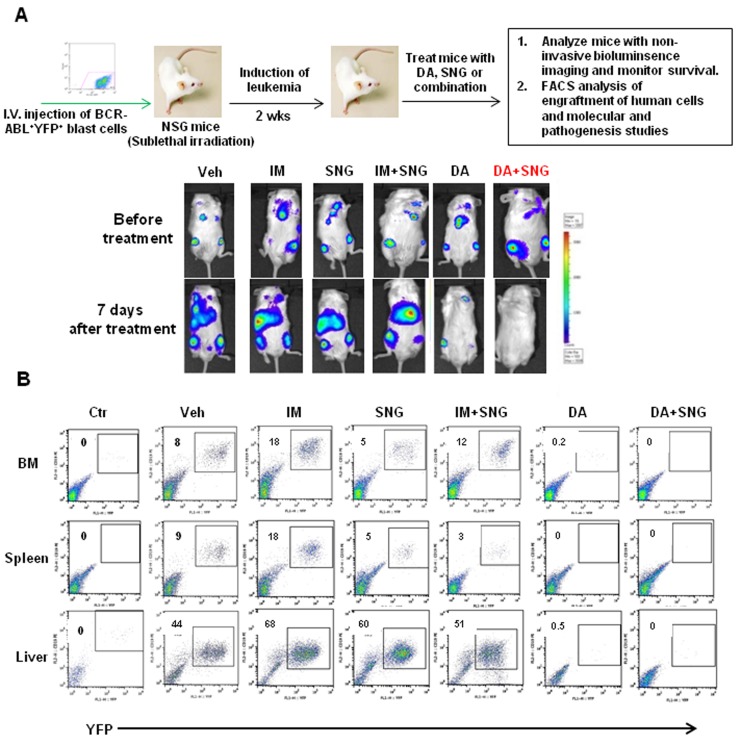
Effects of oral treatment of SNG1135 with DA on the infiltration of human leukemic cells into hematopoietic tissues of mice **A**. Schematic of *in vivo* experiments to assess the efficacy of drug treatments. 2×10^6^ BV173-YFP cells/per mouse were intravenously injected into sub-lethally irradiated NSG mice. Treatment with DA or SNG1153, alone or in combination, was initiated two weeks after transplantation for two weeks. A non-invasive bioluminescent imaging assay was performed before treatment and one week after treatment and representative images from each treatment group are presented before and after treatment. **B**. One mouse from each group was sacrificed and hematopoietic tissues were collected for analysis. FACS profiles of YFP^+^ cells show the level of human leukemic engraftment in BM, spleen and liver.

To evaluate the efficacy of combination treatment in eliminating BCR-ABL+ blast cells with *in vivo* leukemia propagating activity, we utilized a human cell engrafted mouse model, which has been shown to generate a lethal leukemia in NOD/SCID mice [48–50]. Human BV173-YFP^+^ cells carrying a luciferase reporter (2×10^6^/mouse) were intravenously injected into sublethally irradiated NOD/SCID interleukin 2 receptor γ chain deficient (NSG) mice [48–51]. Two weeks after transplantation, mice were treated with vehicle control (propylene glycol), IM (50mg/kg), DA (15mg/kg), SNG1153 (37.5mg/kg), IM plus SNG1153 or DA plus SNG1153 once a day for two weeks by oral gavage. Non-invasive bioluminescent imaging assays after one week of treatment demonstrated that mice treated with either DA alone or DA plus SNG1153 had dramatically lower intensity bioluminescent signals or no detectable signal compared to vehicle, IM, SNG1153 and SNG1153+IM (Figure 6A). The combination of SNG1153 and DA also decreased the previously established leukemia below detection limits (Figure 6A). Seven mice were sacrificed for analysis at 5.5 weeks post-transplant. FACS analysis and histological examination revealed significantly increased engraftment of leukemic cells and extensive infiltration of leukemic cells into BM, spleen and liver of mice treated with vehicle, IM, SNG1153 and SNG1153+IM, while mice treated with DA or DA plus SNG1153 had no detectable engrafted leukemic cells and no infiltration of these cells into hematopoietic organs (Figure 6B & Supplementary Figure 3B).

**Figure 7:**
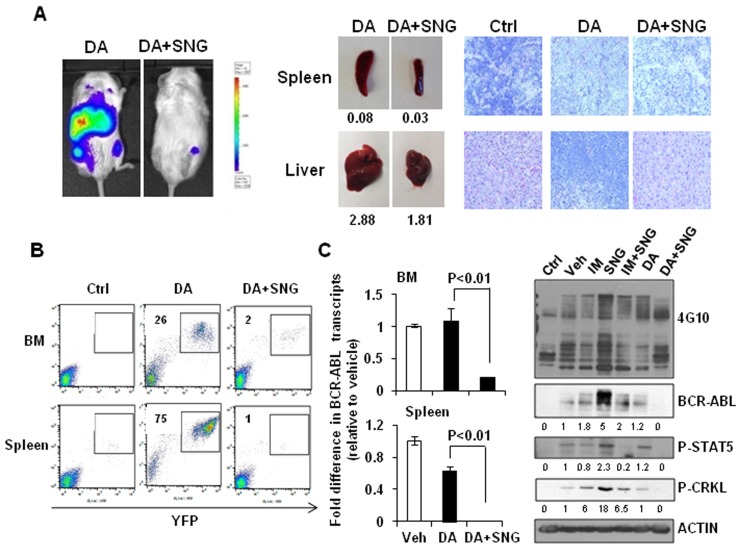
Combination treatment of SNG1153 with DA significantly reduced leukemic cell infiltration in mouse hematopoietic tissues **A**. *In vivo* bioluminescent images, taken eight weeks post-transplantation, of mice treated with DA and DA plus SNG1153. Mice treated with DA or DA plus SNG1153 were analyzed 10 weeks post-transplantation. The weight of spleen and liver, as well as H&E staining of these tissues, is presented. A mouse without any cell injection is presented as a control for H&E staining. **B**. FACS profiles show the engraftment levels of human leukemic cells in BM and spleen. **C**. The fold difference in BCR-ABL transcript levels in BM and spleen compared to a vehicle-treated mouse. Whole protein extracts from BM were analysed by Western blot analysis and probed with antibodies indicated. The densitometry values of protein expression changes are indicated as compared to vehicle control.

**Figure 8:**
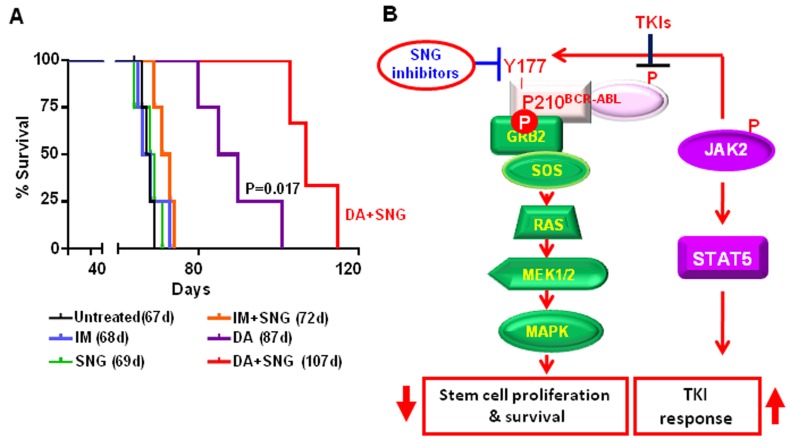
Combination treatment of SNG1153 with DA significantly prolongs the survival of leukemic mice and a model for the mechanism of SNG inhibitors modulating the BCR-ABL-Try177-mediated downstream pathways **A.** The survival curve of leukemic mice treated with inhibitors alone or in combination, as indicated. **B.** Model of the mechanisms by which SNG inhibitors directly inhibit tyrosine phosphorylation of BCR-ABL-Try177 and disrupt its interaction with GRB2 to further inactivate the RAS/MAPK signaling pathway.

The difference in infiltration of leukemic cells in hematopoietic organs between mice treated with DA or DA plus SNG1153 was more pronounced after 10 weeks post-transplantation (Figure 7A). Enlarged spleens and livers were observed in mice treated with DA alone and histological analysis revealed that these organs had more extensive infiltration of human leukemic cells compared to mice treated with DA plus SNG1153 (Figure 7A). FACS analysis demonstrated significantly reduced engraftment in BM and spleen of mice receiving combination treatment compared to DA alone (2 *vs*. 26% & 1 *vs*. 75%, respectively), which correlated to the transcript levels of BCR-ABL in these organs (*p* < 0.01, Figure 7C). In addition, greatly reduced phosphorylation and protein expression of BCR-ABL, p-CRKL and p-STAT5 were observed in mice treated with DA and SNG1153 compared to DA alone (Figure 7C). Importantly, DA plus SNG1153 significantly prolonged survival compared to DA alone (median survival of DA+SNG1153 *vs* DA: 107 *vs*. 87 days, *P* = 0.017), although DA treated mice survived longer than mice treated with vehicle, SNG1153, IM or IM plus SNG1153 (Figure 8A). These results indicate that oral combination treatment with the more potent inhibitors SNG1153 and DA together is more effective than either agent alone, or a combination of SNG1153 with IM, in eliminating BCR-ABL+ blast cells able to generate aggressive leukemia in mice, with significantly enhanced survival of leukemic mice.

## DISCUSSION

Here we provide strong evidence that pre-clinically validated SNG162 and SNG1153 inhibitors, especially more potent SNG1153, effectively target IM-resistant BCR-ABL^+^ blast cells and BCR-ABL-T315I mutant cells. This occurs through a distinct molecular action, which inhibits phosphorylation of the Tyr177 residue at the regulatory domains on BCR and prevents its binding to a key regulator GRB2, leading to reduced activities of the downstream RAS-MAPK pathway (Figure 8B). This suggests a new treatment option for TKI-resistant patients, particularly patients carrying the BCR-ABL-T315I mutation. Specifically, we examined whether SNG inhibitors plus a TKI, to dually inhibit the BCR-ABLTyr177-mediated GRB2-RAS-MAPK pathway in addition to BCR-ABL activity, might be a promising treatment for CML/ALL patients unlikely to respond to TKI monotherapies, as it could more effectively reduce the CML/ALL stem cell burden, and avoid the development of TKI-resistance and disease relapse. Our study on CD34^+^ treatment-naive IM-nonresponder cells provides direct support for this hypothesis. We demonstrated that SNG inhibitors, in combination with a TKI, markedly reduced the output of progenitor colonies and their more primitive stem cells *in vitro*, while these concentrations were much less toxic to primitive healthy BM cells (Figure 4 and Supplementary Figure 3A), providing strong scientific rationale for a therapeutic combination strategy to specifically target TKI-insensitive stem/progenitor cells.

We have also investigated the efficacy of this combination strategy in eradicating BCR-ABL^+^ blast cells *in vivo*, a population with a high frequency of relapse [3, 4]. Indeed, *in vivo* oral administration of SNG1153 and TKI dasatinib significantly eliminated infiltrated leukemic cells to a greater extent in multiple hematopoietic tissues, with statistically significant prolonged survival over TKI or SNG monotherapy (Figure 6-8). Compared with IM, dasatinib is not only a dual SRC-ABL inhibitor, but is also 300-fold more potent in inhibiting ABL kinase *in vitro*, and induces much greater and faster rates of major molecular response [22, 52]. Thus, treatment with the potent TKI dasatinib alone was much more effective at prolonging disease survival than IM alone, and the combination of SNG1153 and dasatinib even more significantly enhances survival of leukemic mice and prevents infiltration of leukemic cells in hematopoietic tissues (Figure 8A). It was noted that the combination of SNG1153 with IM did not result in an *in vivo* synergistic effect, possibly due to the aggressive nature of this leukemia model and much lower potency of IM, compared to DA, for treatment of late stage disease, as observed clinically [3, 4] [22, 52].

Interestingly, it has been suggested that Icaritin and its analogs target abnormal activity of ERα36, an alternative splicing variant of ERα66 with its own promoter, and subsequently inhibit cancer cell growth and induce apoptosis in a variety of cancer cell types [38–41, 44][53, 54]. The biological role of ERα36 and its relationship with BCR-ABL in CML/ALL is not known, but we demonstrated for the first time that ERα36 is highly expressed in BCR-ABL^+^ leukemic cells, including BCR-ABL-T315I mutant cells, and abnormally localizes to cytoplasm and cell membrane of these cells, differing from full-length ERα66 (Figure 1). More interestingly, increased expression of surface ERα36 was found in CD34^+^ IM-nonresponder cells compared to IM-responder cells or CD34^+^ normal BM cells, and knockdown of ERα36 in CML cells increased the sensitivity to IM treatment, while suppression of full length ERα66 did not have an inhibitory effect. These results provide new insights into the critical role of ERα36 in mediation of TKI response/resistance of primitive leukemic cells.

Mechanistically, one of the most important findings of this study is the identification of the inhibitory effects of SNG inhibitors on the BCR-ABL-Tyr177-mediated GRB2-RAS-MAPK pathway, within which SNG inhibitors inhibit phosphorylation of BCR-ABL at the Tyr177 site, which prevents its binding to GRB2 and further reduces phosphorylation of ERK1/2. This observation was demonstrated in multiple BCR-ABL^+^ myeloid and lymphoblastic cells, indicating that it is an important common mechanism contributing to TKI-resistance (Figure 2-5). Since it has been reported that c-ABL can physically interact with ERα66 to form a complex and inhibition of ABL activity sensitizes breast cancer cells to chemotherapy drugs [55, 56], it is possible that ERα36, as its full-length ERα66, interacts with BCR-ABL, contributing to the activation of the BCR-ABL-Tyr177-mediated GRB2-RAS-MAPK pathway and that its inhibition by SNG inhibitors plus TKIs effectively disrupts the BCR-ABL-Tyr177-GRB2 complex and inhibits its downstream pathway (Figure 8B). In addition, the importance of tyrosine phosphorylation of 177 within BCR-ABL in CML pathogenesis has been extensively investigated; in particular, replacing this tyrosine residue with phenylalanine (Y177F) abolishes its transforming activity *in vivo* [10]. Moreover, suppression of GRB2 in BCR-ABL-transduced human CD34^+^ cells greatly inhibits cell proliferation and survival by inhibiting MAPK activation [57]. A recent study also suggests that BCR-ABL-mediated signaling pathways in CML cells are controlled by JAK2 through direct phosphorylation of tyrosine 177 of BCR-ABL [58]. It was interesting to observe that SNG1153 alone completely disrupted the BCR-ABLTyr177-mediated GRB2 protein interaction in BCR-ABL-T315I mutant cells (Figure 3B), but addition of a TKI seems to more effectively dissociate this protein complex in most BCR-ABL^+^ cells (Figure 2, 3 & 5). This is most likely due to BCR-ABL-mediated autophosphorylation of tyrosine 177, since BCR-ABL can phosphorylate itself and several cellular signaling proteins [11, 12]. We therefore hypothesized that while BCR-ABL^+^ cells carrying a T315I mutation prevent binding of a TKI to the ATP-binding pocket of the BCR-ABL TK domain, BCR-ABL TK activity and protein expression are also enhanced in response to this abnormal activity, which in turn further phosphorylates Tyr177 on BCR, to activate the downstream RAS/MAPK pathway. Consistent with our findings, it has been observed that TKI treatment of BCR-ABL cells containing T315I mutation leads to activation of MEK/ERK pathways [59]. Importantly, treatment of cells with MEK inhibitor PD0325901 did not affect phosphorylation of Tyr177, although MAPK activity was completely abolished (by 0.5 μM PD0325901) suggesting that SNG inhibitors directly regulate the phosphorylation status of BCR-ABLTyr177 and reduced p-ERK1/2 activity is likely an indirect, downstream effect (Figure 3C).

Taken together, our study strongly supports a therapeutic role for SNG inhibitors to complement the effects of TKIs by targeting additional key proteins and pathways, leading to more complete disease eradication for CML and BCR-ABL+ ALL patients, particularly those TKI-resistant patients carrying BCR-ABL mutations.

## MATERIALS AND METHODS

### Human cells and suspension cultures

Heparin-anticoagulated peripheral blood (PB) cells were obtained from newly diagnosed patients with CP CML, prior to TKI therapies, who were classified, following IM monotherapy, as IM-nonresponders, based on the European Leukemia Net treatment guidelines (Supplementary Table 1) [45, 46]. Normal bone marrow (NBM) cells came from healthy donors. Informed consent was obtained and procedures were approved by the Research Ethics Board of the University of British Columbia. CD34^+^ cells were enriched utilizing EasySep CD34 positive selection kits as described [50].

CD34^+^ cells were cultured in serum-free medium with growth factors ± TKIs and SNG inhibitors as previously described [50]. Cell lines were cultured in RPMI-1640 media with 10% fetal bovine serum (FBS), 100 u/mL penicillin, 0.1 mg/ml streptomycin and 10^-4^M β-ME.

### Viability and apoptosis assays

After 48 and 72 hours of suspension culture with or without drug treatment, cell viability was assessed using the trypan blue dye exclusion method [50, 51]. Apoptosis analysis was performed using an apoptosis detection kit (BD Biosciences, San Jose, CA). Total apoptotic cell numbers were calculated as the sum of the “early” apoptotic cells (AnnexinV^+^ only) and “late” apoptotic cells (Annexin V^+^/PI^+^).

### Establishment of a stable luciferase expressing BV173-YFP^+^ cell line model

A lentiviral vector MPV-Luc-YFP containing the luciferase report gene was used [60]. Lentiviral production was performed as previously described [50, 51]. To establish a stable luciferase expressing BV173YFP cell line, parental BV173 cells were transduced with lentiviral particles and YFP positive cells were sorted by FACSAria. The sorted cells were maintained in RMPI-1640 supplemented with 10% fetal bovine serum, for further experiments.

### Analysis of drug interactions

Drug interactions were assessed in K562IMR cells after 48 hours of liquid culture in the presence of IM and SNG1153, alone or in combination, as previously described [51]. Briefly, the data were analyzed using constant-ratio drug combinations and the median-effect method of Chou and Talalay. The combination Index (CI) was calculated using CalcuSyn software (Biosoft, Cambridge, United Kingdom). CI < 1, CI = 1 or CI > 1 represent synergistic, additive or antagonistic effects respectively.

### siRNA-mediated knockdown of ERα36 in CML cells

Two FlexiTube estrogen receptor (ER) siRNAs were purchased from Qiagen. Cells were transfected with siRNA using Hiperfect transfection reagents (Qiagen) according to the manufacturer's instructions. Cells were analyzed using a variety of biological and molecular assays after 48 hours of transfection.

### Reagents

Imatinib, nilotinib and dasatinib were obtained from Selleckchem (Houston, TX, USA). SNG inhibitors were obtained from Shenogen Pharma Group Ltd (Beijing, China).

### Immunofluorescence staining and confocal analysis

Cells were prepared on poly-L-lysine coated slides and fixed with 4% paraformaldehyde for 20 minutes at room temperature. The cells were permeabilized and stained with either anti-ERα36 (Shenogen Pharma Corp Ltd) or anti-ERα66 antibody (Santa Cruz Biotechnology), and then incubated with Alexa-488 or Alexa-548 conjugated anti-mouse secondary antibody (Invitrogen). Cells were then counterstained with DAPI and analyzed under a Nikon confocal microscope.

### Immuoprecipitation and western blots

Cells were lysed in PSB buffer and protein concentration was determined as described previously [50, 51]. For immunoprecipitation, cell lysates were incubated with antibodies at 4°C overnight. The immune complexes were incubated with protein G/A bead flurry for another two hours at 4°C. Samples were then heated and separated on 8% SDS-PAGE gels, and membranes were incubated with specific antibodies as listed in supplemental methods. Antibodies used were anti-ABL (8E9; BD Bioscience), anti-phospho-ERK (Cell signaling Technology), anti-GRB2 (Santa Cruz Biotechnology), anti-phospho-Y177 BCR (Cell Signaling), anti-STAT5 (Cell Signalling), anti-phopho-STAT5 (Cell Signaling), anti-phospho-CRKL (Cell Signaling), and anti-phosphotyrosine antibodies (4G10; Millipore).

### Colony forming cell (CFC) and long term culture-initiating cell (LTC-IC) assays

CFC assays were performed as described [50]. Briefly, CD34^+^ CML cells were mixed with methylcellulose medium and IM (5μM), SNG162 (10μM), SNG1153 (5μM), alone or in combination. Colonies were counted after two weeks. For LTC-IC assays, CD34^+^ CML cells were seeded on pre-established, irradiated stromal cells (M2-10B4) in myeloid long-term culture medium (Myelocult medium, STEMCELL Technologies) ± TKIs and SNG inhibitors. Cultures were maintained for six weeks, with weekly half medium changes. Cells were harvested and counted, and CFC assays were performed to obtain LTC-IC-derived CFC colonies.

### Transplantation experiments

2×10^6^ BV173YFP^+^ cells/mouse were injected intravenously into 8 to 10-week-old, sub-lethally irradiated (315 cGy) NOD/SCID-interleukin 2 receptor γ chain deficient (NSG) mice. Two weeks after transplantation, prior to the initiation of treatment, mice were analyzed for engraftment levels of leukemic cells by a non-invasive bioluminescence imaging assay. To perform bioluminescence imaging assays, 50mg/kg D-luciferin was injected intraperitoneally and the injected mice were anesthetized by isoflurane. The signal was detected by an IVIS Lumina II CCD camera system. Mice were then treated with vehicle (propylene glycol), dasatinib (15mg/kg), SNG1153 (37.5 mg/kg), or DA plus SNG1153 by oral gavage once a day for two weeks (5-8 mice per group). Bioluminescence imaging assays were performed at 2, 5 and 7.5 weeks post-transplantation to monitor the engraftment levels of human CML cells in mice from different groups. The level of engrafted CML cells in BM, spleen and liver was analysed by detection of YFP+ leukemic cells by FACS analysis. Liver and spleen from each animal were fixed with 10% (v/v) formalin, paraffin embedded and stained for H&E, for pathology assessment. The injected mice were monitored daily for weight loss and lethargy.

### Statistical analysis

Results are shown as the mean ± standard error of the mean (SEM) for at least three independent experiments. The difference between two groups was compared using two-tailed Student's t-test for paired samples. One way ANOVA was also used, with correction for multiple group comparison, on GraphPad Prism version 6 (http://www.graphpad.com/prism/prism.htm). A *P* value < 0.05 was considered significant. For survival curve analysis, log-rank tests were utilized to compare median survival of mice from different treatment groups.

## SUPPLEMENTARY MATERIALS FIGURES





## References

[1] Sawyers CL (1999). Chronic myeloid leukemia. N Engl J Med.

[2] Goldman JM, Melo JV (2001). Targeting the BCR-ABL tyrosine kinase in chronic myeloid leukemia. N Engl J Med.

[3] Lee HJ, Thompson JE, Wang ES, Wetzler M (2011). Philadelphia chromosome-positive acute lymphoblastic leukemia: current treatment and future perspectives. Cancer.

[4] Jeha S, Coustan-Smith E, Pei D, Sandlund JT, Rubnitz JE, Howard SC, Inaba H, Bhojwani D, Metzger ML, Cheng C, Choi JK, Jacobsen J, Shurtleff SA (2014). Impact of tyrosine kinase inhibitors on minimal residual disease and outcome in childhood Philadelphia chromosome-positive acute lymphoblastic leukemia. Cancer.

[5] Woolfson A, Jiang X, Koschmieder S, Krug U (2001). Targeting the Chronic Myeloid Leukemia Stem Cell: A paradigm for the curative treatment of human malignancies, myeloid leukemia - basic mechanisms of leukemogenesis.

[6] Lugo TG, Pendergast AM, Muller AJ, Witte ON (1990). Tyrosine kinase activity and transformation potency of bcr-abl oncogene products. Science.

[7] Perrotti D, Jamieson C, Goldman J, Skorski T (2010). Chronic myeloid leukemia: mechanisms of blastic transformation. J Clin Invest.

[8] Chu S, Li L, Singh H, Bhatia R (2007). BCR-tyrosine 177 plays an essential role in Ras and Akt activation and in human hematopoietic progenitor transformation in chronic myelogenous leukemia. Cancer Res.

[9] Woessner DW, Lim CS (2013). Disrupting BCR-ABL in combination with secondary leukemia-specific pathways in CML cells leads to enhanced apoptosis and decreased proliferation. Mol Pharm.

[10] Johnson KJ, Griswold IJ, O’Hare T, Corbin AS, Loriaux M, Deininger MW, Druker BJ (2009). A BCR-ABL mutant lacking direct binding sites for the GRB2, CBL and CRKL adapter proteins fails to induce leukemia in mice. PLoS One.

[11] Sattler M, Mohi MG, Pride YB, Quinnan LR, Malouf NA, Podar K, Gesbert F, Iwasaki H, Li S, Van Etten RA, Gu H, Griffin JD, Neel BG (2002). Critical role for Gab2 in transformation by BCR/ABL. Cancer Cell.

[12] Gu S, Chan WW, Mohi G, Rosenbaum J, Sayad A, Lu Z, Virtanen C, Li S, Neel BG, Van Etten RA (2016). Distinct GAB2 signaling pathways are essential for myeloid and lymphoid transformation and leukemogenesis by BCR-ABL1. Blood.

[13] Druker BJ, Guilhot F, O’Brien SG, Gathmann I, Kantarjian H, Gattermann N, Deininger MW, Silver RT, Goldman JM, Stone RM, Cervantes F, Hochhaus A, Powell BL (2006). Five-year follow-up of patients receiving imatinib for chronic myeloid leukemia. N Engl J Med.

[14] Druker BJ, Tamura S, Buchdunger E, Ohno S, Segal GM, Fanning S, Zimmermann J, Lydon NB (1996). Effects of a selective inhibitor of the Abl tyrosine kinase on the growth of Bcr-Abl positive cells. Nat Med.

[15] Gorre ME, Sawyers CL (2002). Molecular mechanisms of resistance to STI571 in chronic myeloid leukemia. Curr Opin Hematol.

[16] Deininger M, Buchdunger E, Druker BJ (2005). The development of imatinib as a therapeutic agent for chronic myeloid leukemia. Blood.

[17] O’Hare T, Zabriskie MS, Eiring AM, Deininger MW (2012). Pushing the limits of targeted therapy in chronic myeloid leukaemia. Nat Rev Cancer.

[18] Gorre ME, Mohammed M, Ellwood K, Hsu N, Paquette R, Rao PN, Sawyers CL (2001). Clinical resistance to STI-571 cancer therapy caused by BCR-ABL gene mutation or amplification. Science.

[19] Jiang X, Saw KM, Eaves A, Eaves C (2007). Instability of BCR-ABL gene in primary and cultured chronic myeloid leukemia stem cells. J Natl Cancer Inst.

[20] Eide CA, O’Hare T (2015). Chronic myeloid leukemia: advances in understanding disease biology and mechanisms of resistance to tyrosine kinase inhibitors. Curr Hematol Malig Rep.

[21] Shah NP, Sawyers CL (2003). Mechanisms of resistance to STI571 in Philadelphia chromosome-associated leukemias. Oncogene.

[22] Shah NP, Tran C, Lee FY, Chen P, Norris D, Sawyers CL (2004). Overriding imatinib resistance with a novel ABL kinase inhibitor. Science.

[23] Weisberg E, Manley PW, Breitenstein W, Bruggen J, Cowan-Jacob SW, Ray A, Huntly B, Fabbro D, Fendrich G, Hall-Meyers E, Kung AL, Mestan J, Daley GQ (2005). Characterization of AMN107, a selective inhibitor of native and mutant Bcr-Abl. Cancer Cell.

[24] Cortes JE, Jones D, O’Brien S, Jabbour E, Konopleva M, Ferrajoli A, Kadia T, Borthakur G, Stigliano D, Shan J, Kantarjian H (2010). Nilotinib as front-line treatment for patients with chronic myeloid leukemia in early chronic phase. J Clin Oncol.

[25] Tokarski JS, Newitt JA, Chang CY, Cheng JD, Wittekind M, Kiefer SE, Kish K, Lee FY, Borzillerri R, Lombardo LJ, Xie D, Zhang Y, Klei HE (2006). The structure of Dasatinib (BMS-354825) bound to activated ABL kinase domain elucidates its inhibitory activity against imatinib-resistant ABL mutants. Cancer Res.

[26] Cortes JE, Kantarjian H, Shah NP, Bixby D, Mauro MJ, Flinn I, O’Hare T, Hu S, Narasimhan NI, Rivera VM, Clackson T, Turner CD, Haluska FG (2012). Ponatinib in refractory Philadelphia chromosome-positive leukemias. N Engl J Med.

[27] Cortes JE, Kim DW, Pinilla-Ibarz J, le Coutre P, Paquette R, Chuah C, Nicolini FE, Apperley JF, Khoury HJ, Talpaz M, DiPersio J, DeAngelo DJ, Abruzzese E (2013). A phase 2 trial of ponatinib in Philadelphia chromosome-positive leukemias. N Engl J Med.

[28] Bhatia R, Holtz M, Niu N, Gray R, Snyder DS, Sawyers CL, Arber DA, Slovak ML, Forman SJ (2003). Persistence of malignant hematopoietic progenitors in chronic myelogenous leukemia patients in complete cytogenetic remission following imatinib mesylate treatment. Blood.

[29] Jiang X, Zhao Y, Smith C, Gasparetto M, Turhan A, Eaves A, Eaves C (2007). Chronic myeloid leukemia stem cells possess multiple unique features of resistance to BCR-ABL targeted therapies. Leukemia.

[30] Corbin AS, Agarwal A, Loriaux M, Cortes J, Deininger MW, Druker BJ (2011). Human chronic myeloid leukemia stem cells are insensitive to imatinib despite inhibition of BCR-ABL activity. J Clin Invest.

[31] Zhang XT, Kang LG, Ding L, Vranic S, Gatalica Z, Wang ZY (2011). A positive feedback loop of ER-alpha36/EGFR promotes malignant growth of ER-negative breast cancer cells. Oncogene.

[32] Wang Z, Zhang X, Shen P, Loggie BW, Chang Y, Deuel TF (2006). A variant of estrogen receptor-{alpha}, hER-{alpha}36: transduction of estrogen- and antiestrogen-dependent membrane-initiated mitogenic signaling. Proc Natl Acad Sci U S A.

[33] Wang ZY, Yin L (2015). Estrogen receptor alpha-36 (ER-alpha36): A new player in human breast cancer. Mol Cell Endocrinol.

[34] Lin SL, Yan LY, Zhang XT, Yuan J, Li M, Qiao J, Wang ZY, Sun QY (2010). ER-alpha36, a variant of ER-alpha, promotes tamoxifen agonist action in endometrial cancer cells via the MAPK/ERK and PI3K/Akt pathways. PLoS One.

[35] Miceli V, Cocciadiferro L, Fregapane M, Zarcone M, Montalto G, Polito LM, Agostara B, Granata OM, Carruba G (2011). Expression of wild-type and variant estrogen receptor alpha in liver carcinogenesis and tumor progression. OMICS.

[36] Wang Z, Zhang X, Shen P, Loggie BW, Chang Y, Deuel TF (2005). Identification, cloning, and expression of human estrogen receptor-alpha36, a novel variant of human estrogen receptor-alpha66. Biochem Biophys Res Commun.

[37] Hong J, Zhang Z, Lv W, Zhang M, Chen C, Yang S, Li S, Zhang L, Han D, Zhang W (2013). Icaritin synergistically enhances the radiosensitivity of 4T1 breast cancer cells. PLoS One.

[38] Wang ZQ, Lou YJ (2004). Proliferation-stimulating effects of icaritin and desmethylicaritin in MCF-7 cells. Eur J Pharmacol.

[39] Tong JS, Zhang QH, Huang X, Fu XQ, Qi ST, Wang YP, Hou Y, Sheng J, Sun QY (2011). Icaritin causes sustained ERK1/2 activation and induces apoptosis in human endometrial cancer cells. PLoS One.

[40] Zhu S, Wang Z, Li Z, Peng H, Luo Y, Deng M, Li R, Dai C, Xu Y, Liu S, Zhang G (2015). Icaritin suppresses multiple myeloma, by inhibiting IL-6/JAK2/STAT3. Oncotarget.

[41] Zhao H, Guo Y, Li S, Han R, Ying J, Zhu H, Wang Y, Yin L, Han Y, Sun L, Wang Z, Lin Q, Bi X (2015). A novel anti-cancer agent Icaritin suppresses hepatocellular carcinoma initiation and malignant growth through the IL-6/Jak2/Stat3 pathway. Oncotarget.

[42] Zhu J, Li Z, Zhang G, Meng K, Kuang W, Li J, Zhou X, Li R, Peng H, Dai C, Shen JK, Gong F, Xu Y (2011). Icaritin shows potent anti-leukemia activity on chronic myeloid leukemia in vitro and in vivo by regulating MAPK/ERK/JNK and JAK2/STAT3 /AKT signalings. PLoS One.

[43] Deutsch E, Maggiorella L, Wen B, Bonnet ML, Khanfir K, Frascogna V, Turhan AG, Bourhis J (2004). Tyrosine kinase inhibitor AG1024 exerts antileukaemic effects on STI571-resistant Bcr-Abl expressing cells and decreases AKT phosphorylation. Br J Cancer.

[44] Tan HL, Chan KG, Pusparajah P, Saokaew S, Duangjai A, Lee LH, Goh BH (2016). Anti-cancer properties of the naturally occurring aphrodisiacs: icariin and its derivatives. Front Pharmacol.

[45] Forrest DL, Jiang X, Eaves CJ, Smith CL (2008). An approach to the management of chronic myeloid leukemia in British Columbia. Curr Oncol.

[46] Baccarani M, Cortes J, Pane F, Niederwieser D, Saglio G, Apperley J, Cervantes F, Deininger M, Gratwohl A, Guilhot F, Hochhaus A, Horowitz M, Hughes T (2009). Chronic myeloid leukemia: an update of concepts and management recommendations of European LeukemiaNet. J Clin Oncol.

[47] Jiang X, Forrest D, Nicolini F, Turhan A, Guilhot J, Yip C, Holyoake T, Jorgensen H, Lambie K, Saw KM, Pang E, Vukovic R, Lehn P (2010). Properties of CD34+ CML stem/progenitor cells that correlate with different clinical responses to imatinib mesylate. Blood.

[48] Shultz LD, Lyons BL, Burzenski LM, Gott B, Chen X, Chaleff S, Kotb M, Gillies SD, King M, Mangada J, Greiner DL, Handgretinger R (2005). Human lymphoid and myeloid cell development in NOD/LtSz-scid IL2R gamma null mice engrafted with mobilized human hemopoietic stem cells. J Immunol.

[49] Dazzi F, Capelli D, Hasserjian R, Cotter F, Corbo M, Poletti A, Chinswangwatanakul W, Goldman JM, Gordon MY (1998). The kinetics and extent of engraftment of chronic myelogenous leukemia cells in non-obese diabetic/severe combined immunodeficiency mice reflect the phase of the donor's disease: an in vivo model of chronic myelogenous leukemia biology. Blood.

[50] Chen M, Gallipoli P, DeGeer D, Sloma I, Forrest DL, Chan M, Lai D, Jorgensen H, Ringrose A, Wang HM, Lambie K, Nakamoto H, Saw KM (2013). Targeting primitive chronic myeloid leukemia cells by effective inhibition of a new AHI-1-BCR-ABL-JAK2 complex. J Natl Cancer Inst.

[51] Lin H, Chen M, Rothe K, Lorenzi MV, Woolfson A, Jiang X (2014). Selective JAK2/ABL dual inhibition therapy effectively eliminates TKI-insensitive CML stem/progenitor cells. Oncotarget.

[52] Kantarjian H, Shah NP, Hochhaus A, Cortes J, Shah S, Ayala M, Moiraghi B, Shen Z, Mayer J, Pasquini R, Nakamae H, Huguet F, Boque C (2010). Dasatinib versus imatinib in newly diagnosed chronic-phase chronic myeloid leukemia. N Engl J Med.

[53] Liu J, Xu Z, Ma X, Huang B, Pan X (2015). Role of ER-alpha36 in breast cancer by typical xenoestrogens. Tumour Biol.

[54] Zhang XT, Ding L, Kang LG, Wang ZY (2012). Involvement of ER-alpha36, Src, EGFR and STAT5 in the biphasic estrogen signaling of ER-negative breast cancer cells. Oncol Rep.

[55] He X, Zheng Z, Song T, Wei C, Ma H, Ma Q, Zhang Y, Xu Y, Shi W, Ye Q, Zhong H (2010). c-Abl regulates estrogen receptor alpha transcription activity through its stabilization by phosphorylation. Oncogene.

[56] Zhao H, Ou-Yang F, Chen IF, Hou MF, Yuan SS, Chang HL, Lee YC, Plattner R, Waltz SE, Ho SM, Sims J, Wang SC (2010). Enhanced resistance to tamoxifen by the c-ABL proto-oncogene in breast cancer. Neoplasia.

[57] Modi H, Li L, Chu S, Rossi J, Yee JK, Bhatia R (2011). Inhibition of Grb2 expression demonstrates an important role in BCR-ABL-mediated MAPK activation and transformation of primary human hematopoietic cells. Leukemia.

[58] Samanta A, Perazzona B, Chakraborty S, Sun X, Modi H, Bhatia R, Priebe W, Arlinghaus R (2011). Janus kinase 2 regulates Bcr-Abl signaling in chronic myeloid leukemia. Leukemia.

[59] Packer LM, Rana S, Hayward R, O’Hare T, Eide CA, Rebocho A, Heidorn S, Zabriskie MS, Niculescu-Duvaz I, Druker BJ, Springer C, Marais R (2011). Nilotinib and MEK inhibitors induce synthetic lethality through paradoxical activation of RAF in drug-resistant chronic myeloid leukemia. Cancer Cell.

[60] Nguyen LV, Pellacani D, Lefort S, Kannan N, Osako T, Makarem M, Cox CL, Kennedy W, Beer P, Carles A, Moksa M, Bilenky M, Balani S (2015). Barcoding reveals complex clonal dynamics of de novo transformed human mammary cells. Nature.

