# Supplementary material for: Targeting BCR-ABL+ stem/progenitor cells and BCR-ABL-T315I mutant cells by effective inhibition of the BCR-ABL-Tyr177-GRB2 complex
Source: Oncotarget. 2017 May 25;8(27):43662–77. doi: 10.18632/oncotarget.18216 (PMC5546432; doi:10.18632/oncotarget.18216)
Supplement: Supplementary file 1 [file oncotarget-08-43662-s001.pdf]

Targeting BCR-ABL⁺ stem/progenitor cells and BCR-ABL-T315I mutant cells by effective inhibition of the BCR-ABL-Tyr177-GRB2 complex

Supplementary Material

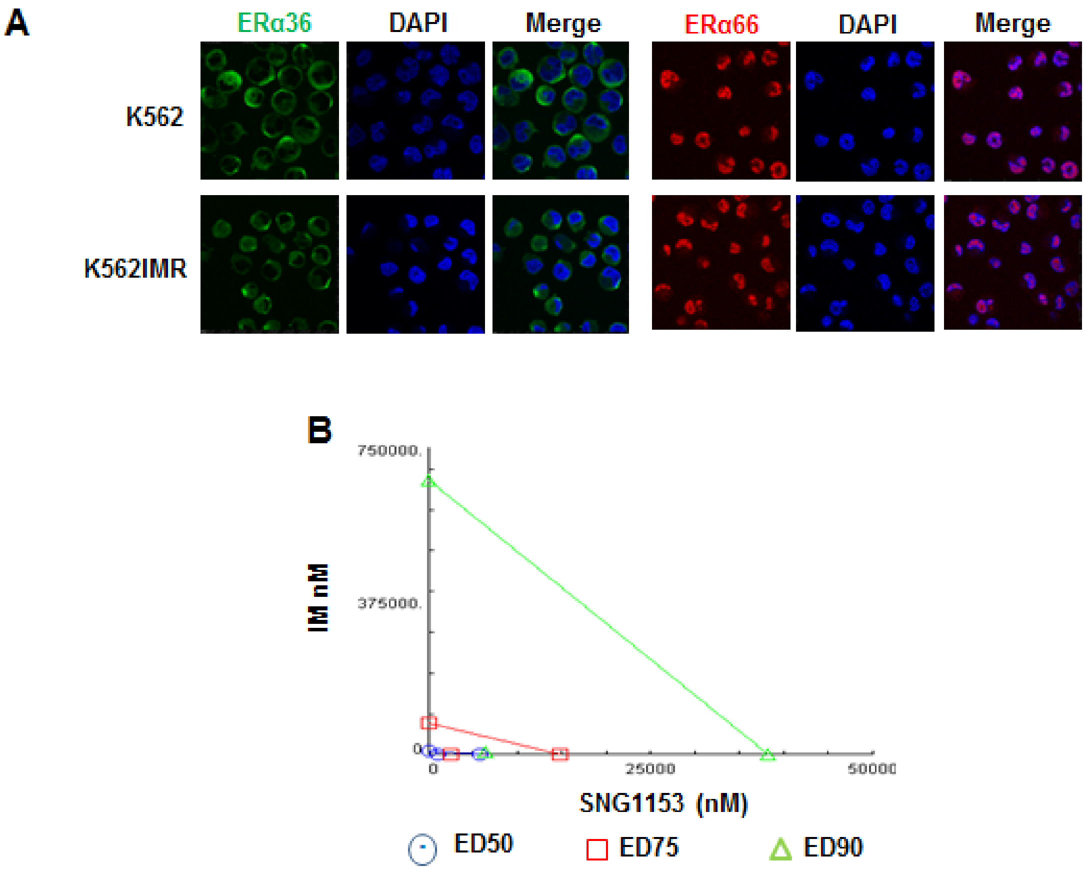

Supplementary Figure 1: Synergistic effects of combination treatment of IM and SNG1153 in CML cells (A) K562 and K562IMR cells were stained with either a specific ERα36 antibody or an anti-ERα66 antibody and analyzed by confocal microscopy. (B) Drug interactions for the combination treatment of SNG1153 and IM in K562IMR cells were assessed by a viability assay after 48 hours of drug exposure. A conservative isobologram analysis for K562IMR cells indicates synergism between IM and SNG1153.

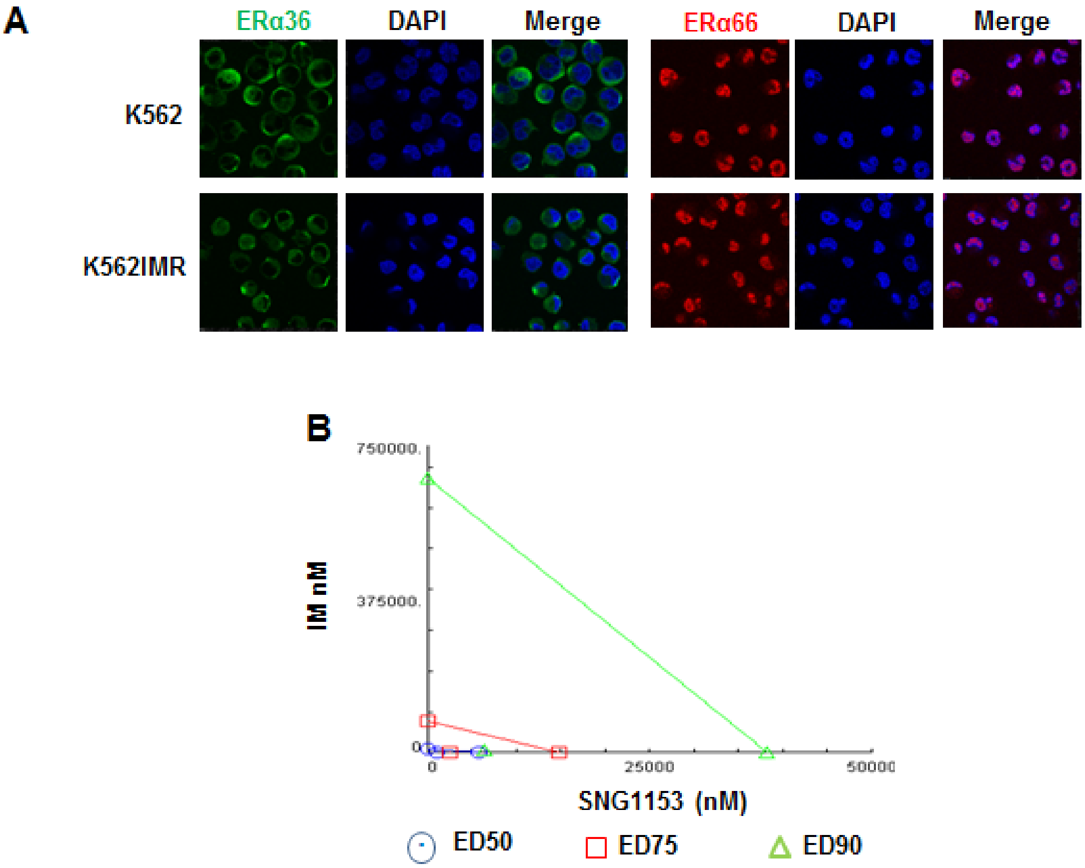

Supplementary Figure 1: Synergistic effects of combination treatment of IM and SNG1153 in CML cells (A) K562 and K562IMR cells were stained with either a specific ERα36 antibody or an anti-ERα66 antibody and analyzed by confocal microscopy. (B) Drug interactions for the combination treatment of SNG1153 and IM in K562IMR cells were assessed by a viability assay after 48 hours of drug exposure. A conservative isobologram analysis for K562IMR cells indicates synergism between IM and SNG1153.

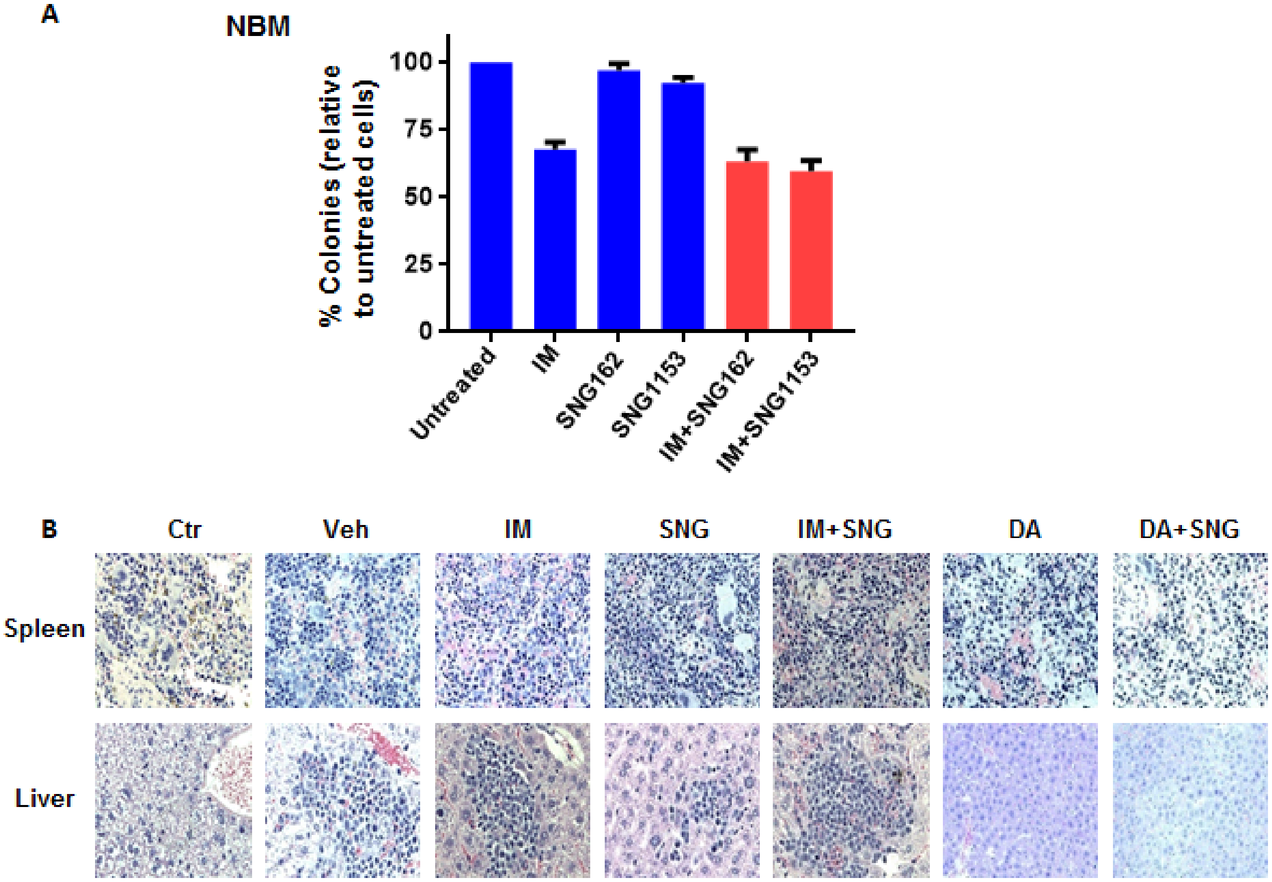

Supplementary Figure 3: No toxicity of SNG162 and SNG1135 on CD34$^+$ healthy BM cells (A) CFC assays were performed on CD34$^+$ bone marrow (BM) cells from normal donors (n=7) with IM (5μM), SNG162 (10μM) or SNG1153 (5μM) alone or in combination. (B) Haemotoxylin and eosin (H&E) staining of spleens and livers from mice with or without treatments, as indicated, 38 days post-transplant.
