# Supplementary material for: Targeting BCR-ABL+ stem/progenitor cells and BCR-ABL-T315I mutant cells by effective inhibition of the BCR-ABL-Tyr177-GRB2 complex
Source: Oncotarget. 2017 May 25;8(27):43662–77. doi: 10.18632/oncotarget.18216 (PMC5546432; doi:10.18632/oncotarget.18216)
Supplement: Supplementary file 2 [file oncotarget-08-43662-s002.doc]

**Supplementary Table 1**: Clinical data for CML patient samples studied

| Patient no | Age at diagnosis | Sex | WBC at diagnosis (10^6^/ml) | Disease stage at IM start | IM response (disease progression)* |
| --- | --- | --- | --- | --- | --- |
| 1 | 45 | M | 392.3 | CP | Non-R |
| 2 | 22 | M | 212.8 | CP | Non-R |
| 3 | 55 | M | 676.6 | CP | Non-R |
| 4 | 66 | F | 492 | CP | Non-R |
| 5 | 21 | M | 202.9 | CP | Non-R (BC) |
| 6 | 48 | M | 140 | CP | Non-R |
| 7 | 43 | M | 282 | CP | Non-R |
| 8 | 39 | M | 105.6 | CP | Non-R |
| 9 | 32 | M | 353.2 | CP | R |
| 10 | 57 | M | 450 | CP | R |
| 11 | 78 | F | 192 | CP | R |

*IM-responders (R) and IM-nonresponders (NR) were classified based on the European Leukemia Net guidelines where IM responders achieved complete hematologic remission within 3 months, major cytogenetic remission within 12 months, and complete cytogenetic remission within 18 months^45,46^. Conversely, IM-nonresponders did not achieve one or more of these response criteria, had evidence of loss of a complete hematologic response or a complete cytogenetic response, or developed blast crisis (BC).
